# Evidence-Based Comparison of Robotic and Open Radical Prostatectomy

**DOI:** 10.1100/tsw.2010.218

**Published:** 2010-11-16

**Authors:** William T. Lowrance, Tatum V. Tarin, Shahrokh F. Shariat

**Affiliations:** ^1^ Department of Surgery (Urology Service), Memorial Sloan-Kettering Cancer Center, New York, NY, USA; ^2^ Department of Urology and Medical Oncology, Weill Cornell Medical Center, New York, NY, USA

**Keywords:** prostate neoplasms, radical prostatectomy, robotic, recurrence, complications, cost, minimally invasive

## Abstract

The rapid adoption of robotic-assisted laparoscopic radical prostatectomy (RALP) has occurred despite a lack of high-quality evidence demonstrating its oncologic advantages, safety, or cost effectiveness compared with open radical retropubic prostatectomy (ORP). This review examines the current literature comparing ORP and RALP, focusing on perioperative, oncologic, functional, and economic outcomes.

## INTRODUCTION

Prostate cancer is the most common urologic malignancy, with more than 2.1 million men in the U.S. estimated to be living with the disease[1,2]. One in approximately every six American men over the age of 50 will receive a diagnosis of prostate cancer in his lifetime[3].

Since the advent of prostate-specific antigen (PSA) screening and with the incorporation of expanded biopsy templates, prostate cancer is now diagnosed in younger men and at earlier stages, resulting in an increased use of local treatment, specifically radical prostatectomy (RP)[4]. Originally introduced by Terrence Millin in 1945 and then significantly refined by Patrick Walsh and colleagues in the 1980s, open radical retropubic prostatectomy (ORP) has long been considered the gold-standard treatment of clinically localized prostate cancer[5,6]. The past 2 decades have ushered in minimally invasive surgical approaches, and urologists have embraced these technological advances and refined approaches for pure laparoscopic and robotic-assisted laparoscopic radical prostatectomy (RALP) with the hope of improving patient outcomes[7,8].

Since more than one-third of the almost 200,000 patients diagnosed yearly with prostate cancer in the U.S. will undergo RP, the effectiveness and side effects of RP have substantial public health implications[9]. However, little data are available for comparing the perioperative and oncologic outcomes of the different RP approaches. In addition, there is limited information on cost differences between ORP and RALP. Unfortunately, a randomized comparison of ORP vs. RALP is unlikely to occur because of difficulties in enrollment in such a surgical trial. Therefore, clinical decision making by patients and physicians can only be made based on case series with relatively small numbers of patients, on retrospective studies, and on a few prospective cohort studies.

The exponential adoption of RALP since the first procedures performed in Germany[10] and the U.S. in 2000[11] has been remarkable and without precedent. A recent population-based analysis showed that minimally invasive RP utilization increased from 9% in 2003 to 43% in 2006[12]. According to unverified estimates from the manufacturer of the da Vinci surgical system (Intuitive Surgical, Sunnyvale, CA), over 80% of RP within the U.S. will be performed robotically in 2010. This drastic shift in RP practice patterns has occurred despite a lack of high-quality evidence demonstrating its oncologic advantages, safety, or cost effectiveness compared with ORP.

As a whole, the urologic literature has been criticized for lacking sufficient high-quality, evidence-based studies. Borawski and colleagues examined published urologic studies in 2005 and found that only 15% of studies were considered level I or II evidence[13]. The debate surrounding the quality of published studies has been especially fervent when comparing RP surgical approaches. In addition, many urologists are unfamiliar with the quality grading and interpretation of the published literature[14]. Two contemporary reviews highlight the shortcomings in studies comparing ORP with RALP and question whether evidence-based medicine is driving RP practice patterns within the U.S.[15,16]. The intent of our review is to provide a critical analysis of ORP and RALP in terms of perioperative, oncologic, functional, and economic outcomes.

## COMPARISON AND DISCUSSION OF ORP AND RALP

### Perioperative Outcomes (Complications, Estimated Blood Loss, Length of Stay)

Perioperative outcomes are commonly reported in studies comparing operative approaches, as they provide unambiguous end points and do not require time-intensive long-term follow-up. Many institutional databases routinely collect information on postoperative complications, estimated blood loss (EBL), and hospital length of stay (LOS), providing clinical researchers with data for comparative studies. The literature comparing perioperative outcomes of ORP with RALP primarily consists of small single-center case series. Population-based datasets, such as the linked SEER-Medicare data, are useful for comparative effectiveness analyses of perioperative outcomes, since they capture a large number of patients across a spectrum of practice settings and geographic locations, with variable surgeon and hospital experience and volume. The advantages of large population-based databases are tempered by the lack of detail on comorbidities, procedure-specific data, etc. Several studies have focused on comparisons of the perioperative complications of ORP and RALP. The reported frequency and the spectrum of complications after RALP and ORP vary widely, making comparisons between series and conclusions difficult. When comparing reported complications between studies, one must carefully consider the specific definition of each complication, the rigor with how the complications were tracked and reported, whether or not a standardized grading system was utilized, and the duration and intensity of follow-up. Each of these factors can greatly influence the data quality and interpretation. Perioperative morbidity or 30-day morbidity, which is reported by many studies, is not sufficient to capture all treatment-related morbidity.

### *Complications*

Two large population-based studies utilizing the SEER-Medicare dataset reported similar findings in regards to postoperative complications after ORP and RALP. Lowrance and colleagues compared the 90-day general medical or surgical complications, and 365-day genitourinary or bowel complications, of 4,697 patients treated with ORP with those of 1,006 patients treated with minimally invasive RP (MRP)[17]. After adjusting for patient and tumor characteristics, as well as year of surgery and surgeon volume, procedure type (ORP vs. MRP) was not significantly associated with a postoperative complication (odds ratio [OR] for general medical or surgical complications: 0.93; 95% confidence interval [CI]: 0.77–1.14; $p = 0.49$; OR for genitourinary or bowel complications: 0.96; 95% CI: 0.76–1.22; $p = 0.74$). In agreement with other studies, these authors found a decreased likelihood of experiencing bladder neck contracture or urethral obstruction in patients treated with MRP compared to those treated with ORP (OR: 0.74; 95% CI: 0.58–0.94; $p < 0.05$). Similarly, in a propensity-score adjusted study of 8,837 men within the SEER-Medicare dataset, Hu et al. found no evidence of difference in overall complications between ORP and MRP[12] (OR for 30-day overall complications: 0.95; 95% CI: 0.77–1.16; $p = 0.58$). It should be noted that both of these studies combined traditional laparoscopic and robotic RPs in their comparative group with ORP. However, the large majority of MRP cases performed in the U.S. are RALP, while only a very small percentage of patients undergo traditional laparoscopic RP. This allows one to consider the MRP data representative of RALP.

In an age- and tumor characteristic–matched comparison of 588 ORPs and 294 RALPs, researchers from the Mayo Clinic found no difference in overall complication rates[18]. One-month complication rates for the RALP group were 8% compared to 5% in the ORP group ($p = 0.064$). One-year complication rates in the RALP group were lower than those in the ORP group (9 vs. 12%); the difference, however, was not statistically significant. The difference in 1-year complications between the groups was primarily due to the significantly higher rate of bladder neck contractures in the ORP group (5 vs. 1%, $p = 0.018$). In a prospective study of 103 RALP and 105 ORP patients (study period: 2006–2007), Ficarra et al. also found similar complication rates[19]. The 30-day unadjusted complication rate for RALP was 10% compared to 11% for ORP ($p = 0.85$). In contrast, in a single-institution retrospective study, Carlsson et al. found a significantly higher overall, unadjusted complication rate for ORP (33%) compared to RALP (16%) (study period: 2002–2007)[20]. Similarly, Tewari and colleagues reported a significantly higher, 30-day unadjusted complication rate for ORP compared to RALP (20 vs. 5%)[21]. While informative, studies comparing operative approaches from single institutions should be scrutinized carefully as their results often hinge on the experience (or volume) of only a few surgeons. If the majority of prostatectomies performed at a given center are performed with an open approach, then one would expect superior outcomes for ORP patients because of the well-known volume/outcome relationship with RP[22]. The same volume/outcome relationship holds true for RALP[17].

In general, the urologic literature demonstrates a similar overall complication rate for ORP and RALP, although there is some minimal disagreement among the studies (Table 1)[15]. In a comprehensive review of the literature comparing open, robotic, and traditional laparoscopic RP, Ficarra et al. found no difference between RALP and ORP (relative risk: 1.33; 95% CI: 0.64–2.74; $p = 0.44$) in their cumulative analysis of overall complications. When the two recently published population-based comparative effectiveness studies[12,17] are considered with the findings from Ficarra and associates[15], there appear to be minimal differences in overall perioperative complication rates between ORP and RALP. The difference in the rate of complications between surgeons seems larger than that between surgical approaches.

## *Estimated Blood Loss and Length of Stay*

EBL and hospital LOS are other commonly compared outcome measures among RP surgical approaches. The widely accepted consensus in the urologic literature is that RALP is associated with significantly less blood loss, lower transfusion rates, and shorter LOS than ORP. These outcomes are important to patients and urologic surgeons for multiple reasons. First, excessive blood loss during RP can jeopardize a patient's cardiovascular health. Furthermore, blood transfusions expose patients to risks of side effects, such as transfusion-associated infections and allergic reactions. Finally, hospital LOS is one of the main determinants in the cost of care.

The data on EBL are consistent throughout the literature. Farnham et al., from Vanderbilt University Medical Center, reported a mean EBL of 664 ml in the ORP group vs. 191 ml in the RALP group ($p < 0.001$)[23]. In a more recent comparison from the same institution, Kordan et al. reported a median EBL of 450 ml in the ORP group compared to 100 ml in the RALP group ($p < 0.001$)[24]. Multiple other

**TABLE 1**
**Perioperative Overall Complication Rates of ORP and RALP**

| Study | Complication Rates* | | p Value |
|---|---|---|---|
| | ORP | RALP | |
| Lowrance[17] | 24% (n = 4,697) | 21% (n = 1,006) | — |
| Hu[12] | 23% (n = 6,899) | 22% (n = 1,938) | 0.31 |
| Carlsson[20] | 33% (n = 485) | 16% (n = 1,253) | — |
| Krambeck[18] | 5% (n = 586) | 8% (n = 294) | 0.064 |
| Tewari[21] | 20% (n = 100) | 5% (n = 200) | <0.05 |
| Ficarra[19] | 11% (n = 105) | 10% (n = 103) | 0.85 |

* Unadjusted overall complication rates. Studies from Lowrance et al. and Hu et al. include patients undergoing traditional LRP and RALP. Because the vast majority of patients in these studies were those undergoing RALP, the patients undergoing traditional LRP and RALP were all classified as RALP patients in this table.

single-institution series have shown lower EBL in patients treated with RALP than in those treated with ORP[19,21,25,26,27,28]. To our knowledge, no study has demonstrated lower EBL in patients treated with ORP compared to RALP.

Although EBL is an important outcome measure, blood transfusion rates are more clinically relevant to patients and the hospital. Kordan and colleagues showed a statistically significant advantage for RALP over ORP in transfusion requirements (0.8 vs. 3.4%, $p = 0.002$)[24]. In reviewing the literature through January 2008, Ficarra and colleagues analyzed six comparative studies and found that ORP patients had a relative risk of receiving a blood transfusion four times higher than RALP patients ($p = 0.01$)[15].

Studies reporting hospital LOS generally favor RALP over ORP. The two comparative effectiveness population-based studies by Hu et al. and Lowrance et al. reported a shorter median LOS for MRP patients (2 vs. 3 days, $p < 0.001$)[12,17]. After adjusting for the effects of patient age, comorbidity, tumor characteristics, year of surgery, and surgeon volume, Lowrance et al. reported a 35% shorter LOS for MRP patients. In addition, increasing surgeon volume was associated with decreasing LOS ($p < 0.001$)[17]. In a comparative study from the Mayo Clinic, 29% of RALP vs. 19% of ORP patients had a 1-day LOS ($p = 0.004$). Nelson et al. showed that over 95% of RP patients can be discharged from the hospital by the end of the first postoperative day[29]. The mean LOS for RALP patients in their study was 1.17 days compared to 1.25 days for ORP patients. Interestingly, their study showed that ORP and RALP patients can be treated with the same clinical care pathway.

## Oncologic Outcomes (Surgical Margins, Biochemical Recurrence)

The priority of most patients undergoing RP is to achieve a cure for their prostate cancer. As such, oncologic outcomes related to RP are of primary importance to both patients and urologists, and represent a valuable measure of success. Surgical margin, biochemical recurrence (BCR), cancer-specific survival, and overall survival rates are all important oncologic end points after prostate cancer treatment, but each has its limitations. Unlike many other malignancies, the natural history of prostate cancer is prolonged and outcomes related to survival may take decades to mature, limiting their practical use. A study initiated today analyzing the overall survival rates of ORP and RALP patients with localized prostate cancer would take well over 10 years to achieve enough events to allow statistical comparison. Such data are not available as of yet. Therefore, we will focus on the more short-term oncologic outcomes such as surgical margins and BCR.

## Surgical Margins

Surgical margins provide urologic surgeons with a quality assessment of their work and help to gauge patients' BCR risk. In addition, surgical margin status is known shortly after the procedure, unlike most other oncologic outcomes. Because the assessment of surgical margin does not require long-term follow-up, it provides a relatively "clean" end point for comparing varying operative approaches, one that is free from the complicated interplay of risk factors that can occur during long-term follow-up. Although surgical margin status has well-known limitations as an oncologic outcome, it has been shown in multiple RP studies to be an independent predictor of BCR that can help to guide the use of adjuvant or salvage therapies[30,31].

In a retrospective comparison of 200 consecutive RALPs with 200 consecutive ORPs, Smith et al. reported a lower positive surgical-margin rate for RALP patients (15 vs. 35%, $p < 0.001$)[32] (Table 2). The authors note that the tumor characteristics for the RALP group at baseline were more favorable than the ORP group. When stratifying according to pathologic stage (pT2), the RALP patients still retained a lower positive surgical-margin rate than the ORP patients (9 vs. 24%, $p < 0.001$). In agreement with other studies, Smith et al. found the apex to be the most common location for a positive surgical margin for both surgical approaches (52% for RALP and 37% for ORP). An update of their experience through 2008 confirmed the lower positive surgical-margin rate for RALP compared to ORP (20 vs. 30%)[33]. Similarly, in a retrospective study of 100 ORP patients and 200 RALP patients, Tewari and colleagues reported a lower positive surgical-margin rate for RALP patients compared to ORP patients (9 vs. 23%, $p < 0.05$)[21]. Another single-surgeon series characterizing the initial experience with RALP reported lower positive surgical-margin rates in the RALP group (22 vs. 36%, $p = 0.007$)[34].

**TABLE 2**
**Oncologic Outcomes in Comparative Studies of ORP vs. RALP: Positive Surgical Margins**

| Study | Surgical Margins | | *p* Value |
|---|---|---|---|
| | ORP | RALP | |
| Smith*[32] | 35% (n = 200) | 15% (n = 200) | <0.001 |
| Krambeck[18] | 17% (n = 588) | 16% (n = 294) | 0.61 |
| Ou[28] | 20% (n = 30) | 50% (n = 30) | — |
| Tewari[21] | 23% (n = 100) | 9% (n = 200) | <0.05 |
| Ficarra[19] | 30% (n = 105) | 34% (n = 103) | 0.97 |
| Kordan*[24] | 31% (n = 414) | 21% (n = 830) | <0.001 |
| Schroeck[35] | 28% (n = 435) | 29% (n = 362) | 0.7 |
| Barocas*[33] | 30% (n = 491) | 20% (n = 1,413) | <0.01 |

* Smith, Kordan, and Barocas studies contain overlapping patients.

Although conceding that the quality of the available comparative studies was not excellent, Ficarra et al., in their cumulative analysis of studies reporting surgical-margin rates for RALP and ORP, found that RALP had a statistically significant advantage over ORP (relative risk 1.58; 95% CI: 1.29–1.94; $p$ <

0.001)[15]. Even when limiting their analysis to prospective studies and those with pathologically localized tumors, RALP continued to obtain a lower positive surgical-margin rate than ORP.

Other single-institution series have reported that surgical-margin rates are similar with RALP and ORP. Krambeck et al. found no difference in positive surgical-margin rates between RALP and ORP(16 vs. 17%, *p* = 0.61)[18]. Their study included nearly 300 RALP patients matched with almost 600 ORP patients; matching was done according to patient age, PSA, Gleason score, clinical stage, and year of surgery. Similarly, Schroeck et al. from Duke University reported no difference in the positive surgical-margin rate of 362 RALP patients and 435 ORP patients (29 vs. 28%, *p* = 0.7)[35]. In a single-institution study, Ficarra and colleagues also found no difference in the positive surgical-margin rates between RALP and ORP (34 vs. 30%, *p* = 0.97)[19]. In their subset analysis of patients with pathologic organ-confined disease, the rate of surgical-margin positivity was the same for both groups (12%).

In conclusion, experienced ORP and RALP surgeons are able to attain negative surgical margins in a high percentage of patients. There are conflicting data on which approach offers superior surgical-margin outcomes, and it is quite possible that surgical-margin status depends more on surgeon than the approach.

## Biochemical Recurrence

Few studies specifically compared BCR rates between RALP and ORP, and those that did are limited by their short follow-up. In a contemporary series by Barocas and colleagues, the authors found no difference in 3-year BCR between surgical approaches (16.5 vs. 16%, log-rank *p* = 0.19)[33](Table 3). Stratification by pathologic stage did not change the findings. While extraprostatic extension, Gleason score 7 or higher, and positive surgical margins were independent predictors of BCR, surgical approach (RALP vs. ORP) was not. Conclusions from this study are limited by the short median follow-up of 10 months.

**TABLE 3**
**Oncologic Outcomes in Comparative Studies of ORP vs RALP: Biochemical PFS Rates**

| Study | Unadjusted 3-Year Biochemical PFS | | Follow-Up | Log-rank *p* Value |
|---|---|---|---|---|
| | ORP | RALP | | |
| Barocas[33] | 84% | 84% | Median ORP = 17 months; RALP = 8 months | 0.19 |
| Krambeck[18] | 92% | 92% | Median ORP = 1.3 years; RALP = 1.3 years | 0.69 |
| Schroeck[35] | NR | NR | Mean ORP = 1.37 years; RALP = 1.09 years | 0.82 |

PFS, progression-free survival; NR, not reported.

In a similar study from the Mayo Clinic with a median follow-up of 16 months, the BCR rate was not different between RALP and ORP patients (8% for both, *p* = 0.69)[18]. Likewise, a comparative study from Duke University with mean follow-up of approximately 1 year found no difference in BCR rates between ORP and RALP after adjusting for the effects of clinical and pathologic features (hazard ratio 0.94; 95% CI: 0.55–1.61; *p* = 0.82)[35].

Using linked SEER-Medicare data, Lowrance et al. found no difference between ORP and RALP in the odds of needing subsequent cancer therapy within 365 days from surgery after adjusting for the effects clinical and pathologic variables (OR: 0.80; 95% CI: 0.60–1.08; *p* = 0.14)[17]. Hu et al. reported similar findings; on adjusted analysis, the rates of subsequent cancer therapy did not differ by surgical approach (MRP 8.2 vs. ORP 6.9 per 100 person-years, *p* = 0.35)[12]. The length of follow-up was a limiting factor in both of these studies.

In summary, the short-term oncologic outcomes between RALP and ORP appear similar. There may be an advantage for RALP in terms of surgical margins, but with limited follow-up, there is no evidence of a difference in BCR rates or in the need for additional cancer therapy.

## Functional Outcomes (Continence, Erectile Function)

The goal for patients undergoing RP is that they be cured of their cancer and retain continence and erectile function. This goal has been termed the “trifecta”[36]. Unfortunately, comparative data for functional outcomes between RALP and ORP are scarce and, to our knowledge, only one study utilized validated instruments to characterize urinary continence and potency after RALP[19]. The lack of uniformity in how functional outcomes are defined and the varying methodology of data collection and analysis further complicate all functional outcome comparisons between RALP and ORP.

### *Continence*

In their prospective, nonrandomized comparative trial, Ficarra et al. found better urinary-continence results for RALP patients compared to ORP patients[19]. Using the International Consultation on Incontinence Questionnaire – Urinary Incontinence (ICIQ-UI) short form, these authors reported 12-month continence rates of 97% for RALP and 88% for ORP ($p$ = 0.001). Patients were categorized as continent if they reported no leak or leaks about once a week or less on the “how often do you leak urine” question. The mean time to continence recovery for RALP patients was 25 days compared with 75 days for ORP patients ($p$ < 0.001). In a single-surgeon study by Ahlering et al., which used an unvalidated questionnaire and defined complete continence as requiring no pads, the authors reported similar continence rates for RALP and ORP (75 vs. 76%)[25]. Using a telephone interview with 120 patients (a subset of the original 300 patients in their study), Tewari et al. also showed that RALP patients had a faster return of urinary continence[21]. The median time to return to continence for the RALP group was 44 days compared to 160 days for the ORP group. Krambeck et al., in contrast, found no difference in urinary continence outcomes between surgical approaches[18]. On an unvalidated questionnaire, patients were considered continent if they had no urinary leakage or required only a security pad. Urinary continence at 1 year was 92% for RALP and 94% for ORP ($p$ = 0.34).

Hu et al. found a significantly higher rate of urinary incontinence diagnosis for MRP compared to ORP (15.9 vs. 12.2 per 100 person-years, $p$ = 0.02)[12]. Nevertheless, when analyzing the need for urinary incontinence procedures, which is a more solid urinary-continence end point, the two population-based studies (Hu et al. and Lowrance et al.) found no significant difference in the need for post-RP urinary-continence procedures[12,17]. Indeed, the reliability of diagnosis codes for characterizing functional outcomes is questionable[37]. Procedure codes provide more concrete end points and seem to result in more unbiased comparisons.

### *Erectile Function*

Comparative data on ORP and RALP regarding erectile-function outcomes parallel the data on urinary incontinence in that they are limited by follow-up time and are restricted to only few centers. Krambeck et al. reported no significant difference in 1-year potency rates between ORP and RALP (63 vs. 70%, $p$ = 0.08)[18]. Potency was defined as erections satisfactory for intercourse with or without phosphodiesterase-5 inhibitors. Tewari et al., in their study using a telephone interview (see above), reported a shorter median time to potency recovery with RALP than with ORP (180 vs. 440 days, $p$ < 0.05). In the only study using a validated instrument to measure erectile function (International Index of Erectile Function-5 [IIEF-5]), Ficarra and associates defined potency as an IIEF-5 score of >17[19].

Limiting the analysis to only those patients receiving bilateral nerve-sparing RP with at least 1 year of follow-up, they found that 49% of ORP and 81% of RALP patients were potent by their definition ($p < 0.001$). Their analysis did adjust for the effects of age, preoperative erectile function, or comorbidity, all of which may have been very different among the two groups.

In conclusion, the literature comparing functional outcomes of RALP and ORP is lacking or inadequate. While there are too few studies with too few patients to draw definitive conclusions at this point, RALP does not seem inferior to ORP with regards to continence and erectile function.

## Cost/Economics

There is also a paucity of data on the economic cost differences between RALP and ORP. Medical expenditures for prostate cancer treatment in the U.S. totaled $1.3 billion in 2000, nearly 30% more than in 1994[38]. Furthermore, RP accounts for approximately half of all prostate cancer expenditures[39]. Given the large number of men with prostate cancer, the effects of treatment have major public health implications, including patient counseling and financial costs. The existing studies comparing the costs of robotic and open radical prostatectomy, however, are limited in their scope, addressing only the costs of the procedure and initial hospital stay[40,41,42]. These studies do not consider subsequent costs from complications, treatment of side effects, or cancer recurrence, which may be substantial in this patient population.

Cost studies by Lotan et al., Mouraviev et al., Scales et al., and Bolenz et al. specifically compared RALP with ORP and reported higher operative costs with RALP, although comparisons were restricted to the costs of the procedure and the duration of initial hospitalization[40,41,42,43]. Scales et al. reported that inpatient costs of RALP are volume dependent and that cost equivalence with ORP is possible at high-volume specialty centers; however, they do not report actual cost data[42]. Bolenz et al. found that the median direct cost was more for RALP than for ORP ($6,752 vs. $4,437, $p < 0.001$)[43]. Economic evaluations of the robotic approach should appropriately account for the da Vinci Surgical System (Intuitive Surgical), which costs approximately U.S.$1.6 million with a maintenance fee of $120,000/year after the first year. The average cost of disposables is approximately $1,500 per case. Bolenz and colleagues estimated the additional purchase and maintenance costs of the robot to be almost $2,700 per patient, based on an annual caseload of 126 cases[43]. This additional "robot cost" is likely an overestimate as this number will shrink with time, especially in high-volume centers.

## CONCLUSION

The rapid adoption of RALP has occurred in the absence of level I evidence showing its superiority or even equivalence to ORP. Unfortunately, the window for conducting a randomized comparison of RP approaches has long passed. The available data point toward advantages in EBL and LOS for RALP compared with ORP. The best available data suggest that there are minimal differences between the surgical approaches in terms of overall postoperative complications. Positive surgical-margin rates may be lower with the robotic approach, but the duration of follow-up in the existing studies is too short to comment definitively on BCR or other important oncologic end points. Likewise, more studies utilizing validated instruments are needed to further characterize differences in functional outcomes between RALP and ORP. It appears that RALP is more costly during the operative period and initial hospitalization than ORP, although the cost difference is likely quite variable depending on surgical volume.

The lack of conclusive data comparing RP operative approaches results in wide variations in practice patterns among urologic surgeons and may ultimately impact the quality of prostate cancer surgical care. At this point, one can argue that RALP is not a cost-effective alternative to ORP for all urologists. However, some urologists may achieve improved outcomes with RALP and therefore tilt the cost-effective balance in favor of the robot. Needless to say, more research is needed in order to determine whether the presumed increase in cost of RALP is justified by improvements in the spectrum of perioperative, oncologic, functional, and economic outcomes.
